# Evaluating the Presence of Disgust in Animals

**DOI:** 10.3390/ani14020264

**Published:** 2024-01-15

**Authors:** Trevor I. Case, Richard J. Stevenson

**Affiliations:** School of Psychological Sciences, Macquarie University, Sydney, NSW 2109, Australia; dick.stevenson@mq.edu.au

**Keywords:** continuity, pathogens, disease avoidance, contamination, great apes, decision tree

## Abstract

**Simple Summary:**

It has been argued that the emotion of disgust is unique to humans. The limited research on this question suggests that animals experience some form of disgust, although the extent remains unclear. To assist researchers in evaluating evidence of disgust in animals, we introduce a disgust decision tree that takes into account alternative explanations for avoidance behavior. This framework is applied to several animal examples. While there is evidence for disgust in animals, we acknowledge that there is a notable difference, even between disgust in humans and other great apes. We explore some of the reasons for this difference and raise the possibility that heightened human disgust may be a recent cultural development.

**Abstract:**

The emotion of disgust in humans is widely considered to represent a continuation of the disease-avoidance behavior ubiquitous in animals. The extent to which analogs of human disgust are evident in nonhuman animals, however, remains unclear. The scant research explicitly investigating disgust in animals has predominantly focused on great apes and suggests that disgust might be present in a highly muted form. In this review, we outline the main approaches to disgust. We then briefly discuss disease-avoidance behavior in nonhuman animals, proposing a set of criteria against which evidence for the presence or absence of disgust in animals can be evaluated. The resultant decision tree takes into account other plausible causes of avoidance and aversion when evaluating whether it is likely that the behavior represents disgust. We apply this decision tree to evaluate evidence of disgust-like behavior (e.g., avoidance of carrion and avoidance of feces-contaminated food) in several examples, including nonhuman great apes. Finally, we consider the large disparity between disgust in humans compared to muted disgust in other great apes, examining the possibility that heightened disgust in humans is a relatively recent cultural acquisition.

## 1. Evaluating the Presence of Disgust in Animals

Disgust is an adaptive emotion that serves the primary function of pathogen avoidance [1,2]. It is a central component of the behavioral immune system [3], which works in tandem with the physiological immune system to protect the individual against disease. In humans, disgust is characterized by an aversive emotional state and a rejection response to pathogen-salient stimuli, which include body products, spoiled foods, certain animals (e.g., worms, rats, and cockroaches), death, certain sexual acts, and unhygienic people [4]. Consistent with the disease-avoidance function of the emotion of disgust, the basic stimuli that have been identified as eliciting disgust in humans have in common that they are pathogen vectors [5], although this approach also acknowledges some plasticity in disgust. For example, sexual arousal can reduce disgust to basic disgust elicitors [6,7]. Beyond such exceptions, however, disgust researchers also include more abstract disgust elicitors (e.g., socio-moral elicitors, which include cheating and deception), which have no obvious link to pathogen threat [4,8].

In this review, we examine the extent to which disgust, an emotion that has been almost exclusively investigated in humans, is present in nonhuman animals. First, we provide a brief overview of the main approaches to disgust, focusing on a process model of disgust [9], which we suggest has the most scope for addressing the continuation of disgust into animals. After discussing the avoidance of toxins and pathogens in animals, we propose a set of criteria for identifying disgust in animals that considers alternative explanations for avoidance. Applying this framework to nonhuman great apes, we focus on the considerably heightened disgust sensitivity in humans compared to other great apes.

## 2. Main Approaches to Disgust

The disease-avoidant approach holds that human disgust is a continuation of the disease-avoidant behavior that is ubiquitous in animals [1,2,10,11]. Thus, behavioral avoidance strategies in animals that reduce the likelihood of contact with pathogens are expected to be driven by a similar disgust-like mechanism. Although understanding the continuity of disgust in animals is germane to understanding the process and function of disgust in humans, the research on disgust has been almost exclusively conducted on humans. Nonetheless, the disease-avoidance approach has become the focus of disgust research over the past few decades and has revealed convergent evidence for the link between pathogen threat and disgust [10]. This has also extended to demonstrating the immunological consequences of disgust [12].

The other influential approach to disgust acknowledges that its evolutionary roots are in aversion to bitter or distasteful food, which is argued to have remained constant over human history and is present in animals. However, this approach holds that disgust was preadapted from the biological system for avoiding bitter tastes (potential toxins) to form disgust to a wide range of elicitors [4]. Accordingly, core elicitors such as feces and other body products have the capacity to produce a disgust response without oral or olfactory sensory input. Importantly, Rozin notes that infants show no rejection of feces, with disgust emerging at about 4–5 years, and argues that disgust is absent in nonhuman animals [13]. Moreover, disgust is held to be driven by beliefs about the nature and the source of the elicitor, which is argued to account for its expansion to encompass a wide range of abstract elicitors such as moral offenses, that pose no pathogen threat. This view holds that human culture plays an important role in the acquisition and expansion of disgust and that, beyond distaste, it is unique to humans [13].

These two perspectives differ in the extent to which pathogen avoidance is primary in the origins of disgust. For the first account, disgust is a biologically evolved part of a pathogen avoidance system that is ubiquitous in animals. Whereas this pathogen-avoidance approach to disgust assumes continuity between the array of pathogen-avoidance behavior in animals and humans, the lack of research on disgust in animals limits our understanding of the nature of this similarity. Disgust to non-pathogen threats in the domains of mate choice and moral judgment are argued to have evolved as separate functions from pathogen avoidance [10]. For the second account, disgust is held to have expanded from the biologically evolved bitter-taste-avoidance system, with disgust reflecting a process of cultural acquisition. Rozin and coworkers [4] argue that this account is consistent with the absence of contamination sensitivity and avoidance of decayed matter in human infants. Moreover, they argue that both contamination sensitivity and avoidance of decayed matter may not be present in other primates [14].

If rejection of bad tastes is included as disgust, then there is clear evidence that it is present in animals. Indeed, primates, rats, and human newborn infants display similar responses to the bitter taste of quinine, including gapes, headshakes, and forelimb flails [15,16]. Although these expressions appear remarkably like disgust reactions in adult humans, Rozin and coworkers [4] argue that this response to bitter substances is toxin avoidance, not pathogen avoidance, and therefore does not represent disgust.

To determine the extent to which disgust is observable in animals, clarity about what is meant by disgust is essential. The two most influential models of disgust [4,10] each derive from theoretical considerations about the function and categorization of disgust elicitors and have generated volumes of research using their respective self-report scales of disgust elicitors. Tybur and coworkers’ [8,10] model, for instance, identifies three domains of disgust, (a) pathogen disgust, serving the function of avoiding contact with sources of disease (e.g., feces and people with signs of infection), (b) sexual disgust, serving the function of avoiding low-value mates (e.g., sexual interest from undesirable person), and (c) moral disgust, serving the function of communicating the condemnation of low-quality others (e.g., theft and deception). Rozin and coworkers [4] identify a different set of domains: core disgust, interpersonal disgust, animal-nature disgust, and moral disgust. Although these can mostly be subsumed by the three domains outlined by Tybur and coworkers [10], there is theoretical inconsistency between these two models in terms of the functions that each domain serves. Moreover, Rozin and coworkers [4] underline the importance of contamination as a central feature of disgust in their model. Accordingly, neutral or acceptable objects (e.g., food) are rendered unacceptable once contacted, even briefly, by a disgust elicitor. The status of contamination for the three-domains model is unclear. Overall, the inconsistencies between the models in the functions, the number of domains of disgust, and the role of contamination make it difficult to use them as a standard for determining the existence of disgust in animals.

### 2.1. A Process Model of Disgust

Recently, a process model of disgust has been proposed that aims to provide clarity about what constitutes disgust [9]. This model has scope for determining the continuation of disgust into animals. According to this model, disgust is generated by four main processes, which are each interlinked by different forms of contamination. We provide a brief outline of the model here, but we refer the reader to Stevenson and coworkers [9] for a thorough discussion.

#### 2.1.1. Pure Disgust

The first of these processes is pure disgust, which is experienced as a direct result of gastrointestinal illness. This pure state of disgust is associated with a feeling of nausea and an intense negative state with a bodily locus. Importantly, pure disgust does not occur in response to the physical presence of an eliciting stimulus. Rather, it may manifest, for example, with delay after ingesting illness-causing food. This form of disgust is proposed to occur in animals that have the capacity to experience gastrointestinal illness and nausea, a capacity that is necessary for all the subsequent processes of disgust.

#### 2.1.2. Somatosensory Disgust

A second process proposed by the model is somatosensory disgust, which is activated by exposure to localized specific gustatory (e.g., poisonous plants), olfactory (e.g., skunk secretion), or tactile (i.e., sliminess and stickiness) cues. These localized cues are proposed to activate dedicated neural circuits that induce a state similar to pure disgust: a feeling of nausea and an intense negative state [9]. However, the locus is specific to the cue (e.g., in the mouth). Since contact has been made by the pathogen threat, the function of somatosensory disgust is to remove or expel the source. Although somatosensory disgust cues signal pathogen threat, unlike pure disgust, there may be some room for flexibility in the disgust response, with this based on the risk of bodily threat posed by the cue [9,17]. This flexibility is consistent with the observed tolerance of certain safe somatosensory disgust elicitors such as sexual contact or consuming certain pungent fermented food (e.g., parmesan cheese). In addition, somatosensory disgust cues are expected to be species-specific, reflecting the relevant pathogen risk to the animal [9].

#### 2.1.3. Anticipatory Disgust

Unlike somatosensory disgust, the third process, anticipatory disgust, is a response to the prospect of contact with a physically present somatosensory disgust elicitor [9]. It occurs only at the sight or sound (no contact) of somatosensory disgust elicitor, and, like pure disgust and somatosensory disgust, it is accompanied by a risk of bodily threat, nausea, and negative affect. Anticipatory disgust comprises aversions that are learned through contact with sensory disgust elicitors. For example, food that visually resembles bad-tasting food is avoided and may evoke nausea, and a negative state. This is compounded by the presence of somatosensory cues (e.g., odor) that usually accompany the perception of anticipatory disgust elicitors. Judging the likely taste of novel foods from prior learning of appearance may be a specific instance of anticipatory disgust—food neophobia. Consistent with this, food neophobia is associated with self-reported disgust sensitivity in humans [9]. There is also evidence of food neophobia in many species [18] including chimpanzees [19].

#### 2.1.4. Simulated Disgust

The final process is simulated disgust, which is produced by imagining somatosensory or anticipatory disgust cues [9]. Much human study of disgust draws upon a capacity for simulation—namely, self-report questionnaires and vignettes requiring respondents to imagine or recall disgust elicitors. Pictures or films of disgust elicitors rely less on imagination to simulate disgust than vignettes or questionnaire items, but they pose no bodily threat. Consequently, they might lead to either anticipatory or simulated disgust. A typical example of a disgust questionnaire item is “You see maggots on a piece of meat in an outdoor garbage pail” [20]. Although imagining such cues can produce a negative affect, imagining is less immediate than being presented with (anticipatory disgust) or touching (somatosensory disgust) maggots. Accordingly, simulated disgust is expected to have less capacity to produce nausea or bodily threat than the other processes of disgust. Given the absence of actual disgust elicitors and minimal risk of bodily threat, simulated disgust is expected to be the one process of disgust that is not present in animals.

#### 2.1.5. Contamination

The model holds that the four processes of disgust are interlinked by three forms of contamination: (a) conditioned taste aversions, (b) physical contamination, and (c) imagined contamination [9]. These forms of contamination govern how neutral objects come to be associated with disgust. In this context, conditioned taste aversions [21] occur when the sick feeling of gastrointestinal illness (pure disgust) is associated with a neutral food cue. The food cue and its sensory qualities then acquire the capacity to produce somatosensory disgust on contact, anticipatory disgust with the prospect of contact, and simulated disgust on imagining the food. Conditioned taste aversions are expected to be a primary way disgust is acquired in animals.

Physical contamination occurs when a neutral object (e.g., food) is contacted by a disgust cue (e.g., feces), rendering the neutral object undesirable. This is expected to be present in animals—especially when signs of the contaminant remain on a preferred food (e.g., feces odor or residue on the food from the contact). However, in humans, the complete removal of the contaminant continues to render the object spoiled [22].

Imagined contamination, whereby both the contaminant and the neutral object are just thought about, has been demonstrated in vignette studies. For example, participants have been asked to indicate their willingness to wear a sweater worn by a murderer, or an infected target, etc. [23]. However, it is difficult to see how this form of contamination could apply to animals.

Overall, the process model of disgust [9] suggests that there is likely to be evidence of pure disgust, somatosensory disgust, and, to a lesser extent, anticipatory disgust in animals. However, simulated disgust, which has characterized most of the human disgust research, is unlikely to exist in animals. Furthermore, there is expected to be evidence for two (conditioned taste aversions, and physical contamination, but not imagined contamination) of the three forms of contamination that link these disgust processes. In the next section, we provide a brief overview of disease-avoidance behavior in a range of taxa and propose a set of criteria that can be used to evaluate evidence for the presence of disgust in animals.

## 3. Disease-Avoidance Behavior in Animals

Composed of 31 phyla, kingdom Animalia contains around 7.8 million species. Any attempt to systematically draw examples from this enormous range would be doomed from inception, both on the grounds of the sheer number and, more importantly, by the lack of knowledge. Disease-avoidance behavior has not been studied in most species. Historically, there have been some important surveys of disease avoidance. By far the best and most extensive comes from Hart’s groundbreaking review [24], as well as from more recent but selective surveys of this literature [11,25]. In this section, the aim is not to provide an extensive overview, but instead to make a single and important point, namely, that across animals that differ radically in brain size (as crudely indexed by the number of neurons in their brains), two basic forms of disease avoidance are consistently encountered. These are avoidance of toxins that typically taste bitter to humans and avoidance of places/entities that signal the presence of pathogens. The key reason for making this our focus is to illustrate why some form of criteria for identifying disgust in animals is required beyond the demonstrable presence of avoidance of toxins and pathogens.

The simplest multicellular animal that we could find that exhibits toxin and pathogen avoidance is *Caenorhabditis elegans* (CE) of the phylum Nematoda. CE is a roundworm, approximately 1 mm long. It is composed of about 1000 cells, of which 302 are neurons. It is, then, by any estimation, a ‘simple’ organism. CE feeds on bacteria, and it is attracted to its prey by certain bacterial metabolites, detected via chemosensory receptors. However, certain bacteria are toxic to CE by either contact or ingestion, and so it has an innate capacity to avoid these pathogenic bacteria [26]. This avoidance is mediated by chemosensory cues. Not only does CE avoid pathogenic bacteria, but it also avoids water-soluble toxins that taste bitter to humans. This, again, is mediated via the animal’s chemosensory system [27].

The phylum Arthropoda represents a step change in neural complexity. In respect to disease avoidance, three different groups of organisms have been studied in this phylum. One group is the social insects. Using the honeybee (e.g., *Apis mellifera*) as a representative example, it has around 1 million neurons. Another group is the fruit flies, particularly *Drosophila melanogaster*, which has around 150,000 neurons. The final group is the lobsters, which are often used in chemosensory research and have around 100,000 neurons. Social insects have a vast and diverse array of disease-avoidant behavior [28]. This includes specialized corpse and feces removal from the nest; nest entry prevention to infected conspecifics; social exclusion of infected conspecifics; social structures that limit animals most at risk of infection from contacting those least at risk; walling off infected individuals/nest areas; and the abandonment of infected nests [28]. Pathogen avoidance is also observed in fruit flies and lobsters. Fruit flies show an innate aversion for egg laying on carnivore feces that contain pathogenic bacteria, with this response mediated by chemosensory cues (probably phenol) [29]. Caribbean spiny lobster, *Panulirus argus*, avoid conspecifics infected with the contagious PaV2 virus, with this avoidance also mediated by chemosensory cues [30]. Turning to the avoidance of toxic substances that humans find bitter tasting, this has been observed in *Drosophila melanogaster* [31] and in many Arthropoda species [32]—although some are clearly tolerant to toxins if they form part of their routine diet (e.g., tobacco moth, *Manduca sexta*).

The most extensively studied phylum regarding disease avoidance is Chordata. It comprises fish, amphibians, reptiles, birds, and mammals, the last mentioned having the largest brains of any animal, ranging from 200 million neurons in the rat to 250 billion in the elephant brain (86 billion for humans). Hart’s [24] review identified two major forms of disease avoidance, with most of the evidence being from mammals. The first concerned macroparasites, which are behaviorally managed via a combination of grooming [11] and fecal avoidance [33]. Grooming is widely observed in mammals, and, interestingly, often involves the ingestion of ticks and fleas that are caught when either grooming oneself or a conspecific. This might explain the lesser disgust observed in humans towards these ectoparasites, relative to endoparasites [34]. Turning to avoidance, this almost always centers on conspecifics’ feces, presumably to minimize ingestion of endoparasitic larvae. This type of avoidance behavior manifests in different ways. It can be seen in carnivorous mammals who do not defecate in or near their dens, in elephants who will not drink from fecally contaminated water, and in many herbivores—reindeer, wallabies, dik-dik, etc.—who avoid consuming forage that is fecally contaminated [11,33,35,36].

The second major category of disease avoidance in mammals is directed at microparasites. A range of different behavior has been observed that are often species-specific, but which all serve to minimize contact with agents or environments that harbor pathogens [24]. This can include social exclusion (i.e., avoiding infection sources), post-copulatory grooming (i.e., removing contaminants), wound licking (i.e., removing contaminants), and disease-avoidant behavior directed at infants (e.g., cannibalism of a sick infant)—with much of this behavior documented in laboratory rats [37]. Of the empirically studied examples of both micro- and macroparasite avoidance (e.g., avoidance of sick conspecifics and avoidance of fecal-contaminated water), chemosensory cues, again, seem to be particularly important [36,37].

As we described for the examples from the phyla Nematoda and Arthropoda, members of the Chordata, and especially mammals, also display avoidance of toxins that taste bitter to humans [38]. While this is moderated by eating habits, such that herbivores tending to be more tolerant of these toxins than carnivores, avoidance is still observed [38]. Another interesting observation, and one that has primarily emerged from studies of rodents, concerns the distinction between avoidance of and aversion to a food [39]. Adult rats fed a novel food, accompanied by lactose, which they cannot digest, experience gastrointestinal discomfort (cramping/bloating) and come to avoid this food [40]. Adult rats that experience a novel food accompanied by the nausea-producing agent lithium chloride also come to avoid this food. However, rats that have experienced nausea with a food show the same signs of orofacial rejection, as seen when they taste bitter compounds. This orofacial rejection is not seen with foods paired with lactose. This important distinction illustrates how avoidance can come in different forms.

This brief survey indicates that, across animals that vary in brain size from 300 to 250,000,000,000 neurons, there is a striking commonality of function, with the avoidance of chemical toxins and pathogens. For less complex species like *Caenorhabditis elegans*, fruit flies, or honeybees, avoidance is likely triggered directly via chemosensory cues and reflexive neural circuits, without the emotion of disgust. However, for complex species that have the necessary capacity to have a nausea-like experience in response to gastrointestinal disturbances (pure disgust), the emotion of disgust is likely to be present.

### A Capacity for Nausea

Determining whether a species is susceptible to the subjective state of nausea [41] and, therefore, possesses the capacity for disgust is difficult. The ability to vomit, which occurs in most vertebrates, seems a useful criterion. Stimuli such as cytotoxic drugs and anti-emetics have been used to investigate emesis and nausea in animal models [42], including in the cat, dog, ferret, guinea pig, hamster, monkey, mouse, rat, and shrew [42,43].

Some non-emetic models (e.g., rats) are also thought to demonstrate behavior that is suggestive of nausea [42]. Conditioned taste avoidance of a food previously paired with an emetic is used to imply the presence of unpleasant nausea-like sensations [43]. Another indicator is pica, or ingestion of non-foods (e.g., kaolin clay dilutes or binds toxins, reducing their adverse effects), which increases in rats with increases in toxins [42].

Although the capacity for nausea among various taxa remains largely unknown, the limited research suggests that nausea-like sensations likely exist in higher mammals. Using similar experimental procedures, research may uncover that the capacity for nausea-like sensations also extends to birds, reptiles, fish, and amphibia. However, for disgust to be present, regardless of the process of disgust (i.e., pure, somatosensory, anticipatory, or simulated), the species must have the capacity to experience nausea-like sensations in response to gastrointestinal disturbances.

Even in mammals with the capacity to experience nausea-like sensations, however, avoidance of potential pathogen sources can come in different forms. To determine, then, if disgust may be present in animals requires a more elaborate scheme, as we outline next.

## 4. Criteria for Evaluating the Presence of Disgust in Animals

**Figure 1:**
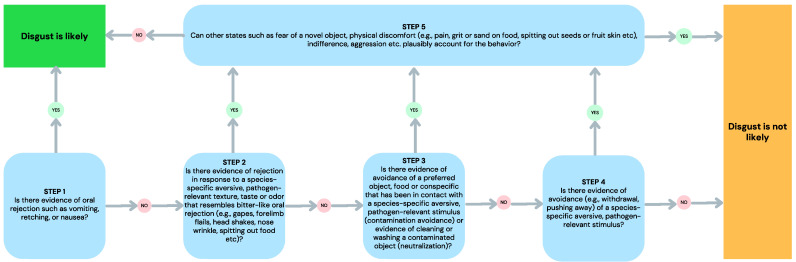
A decision tree for imputing disgust in animals that are susceptible to gastrointestinal illness and have the capacity to experience nausea.

Based on the assumption of continuity inherent in the pathogen-avoidance approach to disgust, we suggest that disgust can sometimes be imputed in animals where avoidance responses to pathogens, contaminants, or toxins are evident [37]. However, avoidance also results from other states (e.g., fear and disinterest). Thus, the stimulus and the context must be considered when evaluating the likelihood that an animal’s avoidance of a stimulus is motivated by the emotion of disgust. Further, there is also a wide variation between species in the ability to (a) display behavior that is indicative of disgust (e.g., vomiting is limited to emetic species) and (b) tolerate and actively seek out pathogen sources (e.g., vultures and rotting flesh). In acknowledgment of the difficulty of imputing disgust in animals, and in drawing on the insights provided by the recent process model of disgust [9] outlined above, we offer the following decision tree (Figure 1) that can be used to decide whether an animal’s behavior is likely to indicate disgust.

Consistent with the disease-avoidance account [1,2], the decision tree is based on the assumption that there is continuity of disgust in other species. Thus, pure disgust is expected to be apparent in any species that is susceptible to gastrointestinal illness, with the capacity to experience nausea. The reference to species-specific pathogen threats acknowledges that different species have evolved specific physiological and immunological defenses that make them more resistant to certain gastrointestinal illnesses. For example, certain scavenger species such as vultures [44] have evolved low gastric pH that can kill many harmful bacteria, providing some level of protection against gastrointestinal infections. Importantly, the elicitor approach to disgust, commonly applied to humans, is inadequate when it comes to determining disgust across species—a rotting animal carcass is a nutritious opportunity to one species, but potentially lethal to another.

The first step of the decision tree recognizes the central role that gastrointestinal illness and nausea serves in the process model of disgust [9]. Evidence of vomiting, retching, or nausea without the physical presence of an eliciting stimulus represents pure disgust in animals that have the capacity to experience gastrointestinal illness. A response to contact (e.g., in the mouth) with an eliciting stimulus would represent somatosensory disgust if the eliciting stimulus was known to be a species-specific aversive taste, odor, or texture. This might also occur when a novel food that has sensory qualities of a known disgust elicitor is sampled in the mouth. Vomiting, retching, or nausea occurring at the sight or sound (no contact) of a disgust elicitor would suggest anticipatory disgust. However, in naturalistic conditions, the active avoidance of the elicitor would be a more likely strategy.

The second step recognizes that there can be other rejection behavior (e.g., head shakes, forearm flails, gapes, spitting, etc.) that suggest gastrointestinal illness or somatosensory cues that invoke nausea. This acknowledges that disgust can be apparent in the many non-emetic species, such as rodents [37]. Rejection responses that do not resemble bitter-like oral rejection in that species, as in the absence of orofacial rejection observed in rats presented with lactose-paired food described above [40], are unlikely to result from revulsion. As for the first step, (anticipatory) rejection behavior occurring at the sight or sound (no contact) of a disgust elicitor would likely be unnecessary because it is effectively addressed by the animal engaging in avoidance. The disgust decision tree requires consideration of other potential causes (e.g., fear, spitting out seeds, etc.) of the rejection behavior at Step 5.

The third step acknowledges that objects (e.g., food), which might otherwise have a positive valence, can become contaminated by contact with a disgust cue (e.g., feces), and then be avoided or subjected to removal of signs of the contaminant (neutralization). If there are no other plausible reasons for this at Step 5 (e.g., removal of sand or grit from food), then disgust is likely. We expect that contamination will be limited to physical, rather than imagined contamination in animals.

The fourth step concerns the realm of active avoidance of species-specific pathogen-relevant stimuli. However, as discussed above, active avoidance may represent many other influences and causes (e.g., indifference or fear). Consequently, at Step 5, the disgust decision tree requires the elimination of alternative plausible explanations for the observed avoidance behavior to be considered disgust.

Based on the process model of disgust, the disgust decision tree offers a way of assessing the process of disgust that may be occurring in a particular species, while allowing for flexibility in species-specific variation in responses and pathogen threats. Importantly, the decision tree also requires an evaluation of the possibility that such disgust-like behavior as rejection, avoidance, and neutralization might occur for other plausible reasons (e.g., fear, discomfort, and indifference). We note again that simulated disgust is unlikely to be present in nonhumans, which we discuss further below.

### 4.1. Applying the Decision Tree

Whereas clear evidence of pure disgust comes from signs of gastrointestinal illness (e.g., vomiting and nausea), rejection and avoidance might represent other causes. It is this ambiguity that the disgust decision tree aims to reduce. Below, we apply the disgust decision tree to an example of avoidance of carrion and avoidance of contaminated food.

#### 4.1.1. Avoidance of Carnivore Carcasses

Carrion represents a highly nutritional reward to scavenger species. However, carnivore carrion exposes the scavenger to the risk of meat-borne parasites [45]. Fulfilling Step 4 (avoidance of species-specific aversive, pathogen-relevant stimulus), Gonzálvez and coworkers [45] observed that red foxes tended to avoid foraging on carnivore carcasses—especially carcasses of conspecifics. Consistent with this avoidance representing disgust, rather than another cause (e.g., fear; Step 5), where consumption of the carcass did occur, it was after a delay of days or weeks from detection. Although this delay reduces the nutritional value of the meat, the risk of meat-borne parasites reduces as the carcass decomposes. Moreover, the late discovery of decaying carcasses by red foxes was associated with a lower consumption delay. By comparison, ungulate carcasses, which pose a low pathogen risk, were usually consumed rapidly. Overall, this example of avoidance of carnivore carrion by red foxes suggests that it is driven by anticipatory disgust.

However, there are other instances, where scavenger avoidance of carnivore carrion does not represent disgust. In one example, wild boar displayed fight and flight (fear) responses on discovering carcasses of its main predator, the gray wolf [46]. No such responses were evident toward fox (a non-predator) carcass sites. Similarly, a group of long-tailed macaques were observed to abruptly change their direction of travel and display vigilance behavior in the proximity of a decaying dog carcass, which is consistent with a fear of predation, rather than disgust [47]. Thus, observed carcass avoidance (Step 4) was qualified by predator fear, not disgust (Step 5).

#### 4.1.2. Avoidance of Contaminated Food

A preferred food that has signs of a contaminant that poses a pathogen threat might be avoided, representing somatosensory or anticipatory disgust (Step 3). In one study [48], bonobos were simultaneously presented with three slices of apple. One slice was clean, while the other two were coated with either soil or feces. While the clean slice was consumed more often than the contaminated slices, there were no reliable differences in the consumption of the pathogen-relevant feces-contaminated slices and the soil-contaminated slices. Consistent with Step 5 of the disgust decision tree, avoidance of the contaminant was not specific to pathogen threat in this instance--the same avoidance was observed for the non-feces-contaminated slices. These researchers also found that bonobos showed no differences in the consumption of feces-contaminated and clean banana slices when visual signs of the contaminant were removed in front of the bonobos [48]. This is a more stringent test of physical contamination avoidance and suggests that, for bonobos, once the overt signs of feces contamination are removed, the food ceases to be contaminated. Interestingly, this form of contamination is a main feature of disgust in humans, where even young children will reject a drink that was previously contacted by an insect [49]. Nonetheless, the experimental approach that Sarabian and colleagues have taken to investigating disgust in great apes and other primates holds great promise for identifying the extent to which processes of disgust are relevant in animals.

### 4.2. Disgust in Great Apes

The disgust decision tree is intended to inform investigations of disgust in a wide range of animals—any that have the capacity to demonstrate gastrointestinal illness and nausea. However, given that the research on disgust has been conducted almost exclusively on humans, the extent to which the different processes of disgust and contamination are present in great apes—the closest living relatives of humans—is of special interest, with the potential to reveal insights into the origins of the complex nature of human disgust [50].

So far, the research on disgust in great apes has been limited, but the presence of pure disgust with signs of gastrointestinal illness such as vomiting, and somatosensory disgust, such as the rejection of unpleasant food from the mouth, is not contentious. In a broad-scope survey of great ape researchers, fieldworkers, and keepers, reports confirmed that great apes (mainly chimpanzees) expel bad-tasting food and withdraw from pungent odors (somatosensory disgust) [50]. Moreover, the removal of feces from the point of contact was commonly observed. For example, one respondent made the following observation of common chimpanzees in captivity:

I saw an adult male step in feces and then pick up a stick to wipe it off his foot. Usually, though, I see the chimps vigorously shaking a body part until the offending waste is shaken off (common chimpanzees in captivity (p. 249, [50]).

This rejection of a pathogen-relevant texture and odor is consistent with Step 2 of the disgust decision tree, yet it is possible that contact with the feces elicited discomfort rather than disgust (Step 5).

There were also reports of avoiding or neutralizing (washing or wiping) preferred foods when they have been contaminated (physical contamination) and avoiding or neutralizing contact with conspecifics’ body products. The reports indicated that food contaminated with soil was often brushed off, but food contaminated with feces tended to be avoided altogether. Avoiding and neutralizing contaminated food is consistent with the requirements of Step 3 in the disgust decision tree. However, the more frequent avoidance of feces-contaminated food and fewer attempts to neutralize it compared to soil-contaminated food suggests that feces represents a disgust cue rather than mere physical discomfort (Step 5).

A further example from a respondent observing Eastern chimpanzees in the wild suggests that feces has the potential to deter male chimpanzees from a copulation opportunity:

I have also seen a female have feces all over her back. She repeatedly and vigorously rubbed her back against trees and wiped her back with leaves. She was in full oestrus at the time, and several males solicited her and approached her for copulation. Upon seeing the feces on her back, ALL males recoiled and quickly moved away (p. 249, [50]).

Although this example is highly suggestive of disgust-driven contamination avoidance (Step 3), more information would be required to rule out other possible causes (Step 5).

Perhaps the most surprising finding to emerge from our broad-scope survey of disgust in great apes was reports of behavior that is contradictory to the presence of disgust. Specifically, great apes tended to be indifferent to contact with blood, mucous, semen, and sick conspecifics. Indeed, sick conspecifics were often approached and groomed. Further, coprophagy (usually confined to the animal’s own feces) was frequently reported—reingestion softens seeds and difficult-to-digest foods and is thus an important source of nutrients [51]. Nonetheless, consuming feces represents a marked departure from disgust in humans.

Whereas avoiding rotting carcasses was common in great apes, there were also instances where this was strikingly absent. For example, there was a report of mountain gorillas in the wild resting close to a dead duiker, seemingly indifferent to the smell. There was also another report of cannibalism of a rotting infant carcass observed in bonobos in the wild. Overall, the findings of our survey [50] and of other recent studies of great apes [25,48,52,53] point to evidence of disgust in great ape species. However, despite being host to many of the same diseases as humans [54], disgust in great apes is muted. This attenuated disgust is apparent for somatosensory and anticipatory disgust and for the process of physical contamination. Moreover, there has been no evidence consistent with the presence of simulated disgust or imagined contamination in great apes.

## 5. Heightened Disgust in Humans

One question that arises from observations of muted disgust in great apes and other animals is why human disgust is so exaggerated. Research on disgust in humans documents an extensive range of disgust elicitors from basic stimuli, such as feces, to abstract social-moral elicitors [4,5,8,55]. We argue that this expansion of disgust in humans to a wide range of elicitors reflects the additional process of simulated disgust and the capacity to imagine contamination [9]. Moreover, the cognitive capacity of humans to imagine contamination, which has been demonstrated in hunter-gatherers [56], has been suggested to represent an important developmental milestone in the emergence of (anticipatory and simulated) disgust in children [4,14,57,58]. However, there may be several additional candidates to explain heightened disgust in humans and we briefly consider these factors below. We suggest that although these factors might have provided the basis for heightened disgust compared with other great apes, exaggerated disgust may be a relatively recent development.

### 5.1. Diet

The move to a greater dependence on carnivory in the diet of *Homo* ancestors is thought to have played an important role in the emergence of heightened disgust in humans [59] and in the evolution of human cognition more generally [60,61]. The benefit of a diet high in meat is that it provided a concentrated source of protein to meet the high energy requirements of the large human body size and brain, reducing the time required foraging [62]. In terms of disgust, the consumption of meat, especially scavenging decaying meat, was also accompanied by a greater risk of gastrointestinal illness [63]. Thus, the emergence of heightened disgust towards signs of contaminated meat would have been beneficial in an omnivore that has increased its dependence on meat but does not have the evolved meat-borne pathogen protections possessed by carnivorous scavengers [44,64]. The comparatively low levels of meat consumption and lack of meat scavenging in nonhuman great apes [65] pose little pathogen threat from meat and are consistent with the muted disgust in these species.

### 5.2. Agriculture and Large Communities

Another factor that may have contributed to heightened disgust in humans is the emergence of agriculture and large communities. Although infectious disease is an important source of natural selection for all great ape species, the emergence of agriculture and increases in population density in humans from about 11,000 years ago saw the generation and proliferation of transmissible and virulent human crowd diseases [66,67]. Moreover, the domestication of animal herds provided a reservoir for the transfer of zoonotic diseases [68]. While this is only a short time in evolutionary terms, there is evidence that there has been rapid genetic evolution since this period, including in genes responsible for pathogen response to crowd diseases [69]. Thus, the emergence of agriculture and increases in population density would have posed a sharp selection pressure on Neolithic humans and may have heightened the existing behavioral defense against pathogens—disgust.

Together, the cognitive capacity to appreciate contamination, the increased dependence on a meat diet, and the selective pressure of infectious diseases associated with population increases and animal domestication provided fertile ground for heightened disgust in humans compared with other great apes. Specifically, these conditions may have set humans apart from other great apes in terms of developing the capacity for simulated disgust and imagined contamination. However, we suggest that contemporary exaggerated disgust sensitivity to the diverse range of elicitors that is evident from the research literature may reflect a relatively recent cultural development.

### 5.3. Non-Western Disgust

Even with the capacity for imagined contamination [56] and, presumably, simulated disgust, there is evidence from ethnohistoric accounts that, in many small-scale, non-Western societies, putrid meat (and maggots) was not only tolerated but also often a preferred meat preparation [70,71]. The advantage of putrification is that, like cooking, it softens and pre-digests meat. Further, putrid meat also reduces the loss of vitamin C [72]. Contrary to the idea that rotting meat poses a significant pathogen threat, there was little evidence in these accounts that the consumption of putrid meat and maggots led to illness. Speth [72] argues that evidence of the consumption of putrid meat and fish in many small-scale non-Western societies is consistent with disgust being culturally determined.

While the ethnohistoric accounts provide compelling evidence of muted disgust in many small-scale societies and suggest this may well have been the case for ancestral humans, we suspect that there might also have been certain proscriptions in these societies regarding putrefied meat consumption. That is, there may have existed exclusions or limits about which type of putrid meat or fish could be consumed. If so, it would reflect a similar modulation of disgust observed towards certain delicacies such as pungent-smelling cheeses (e.g., Casu Marsu, a rare Sardinian cheese that contains living fly larvae [73]) or slimy, raw oysters, and other elicitors in Western cultures [9]. The existence of such exceptions, especially when they are necessary for survival, does not negate the existence of disgust. As an example, coprophagy was frequently observed in our survey of great apes [50], but it usually involved selective picking of seeds from the ape’s own feces, which aids the re-digestion of tough seed husks. Despite this functional coprophagy, aversion to contact with feces was otherwise commonly reported.

### 5.4. A Western Culture of Cleanliness

**Figure 2:**
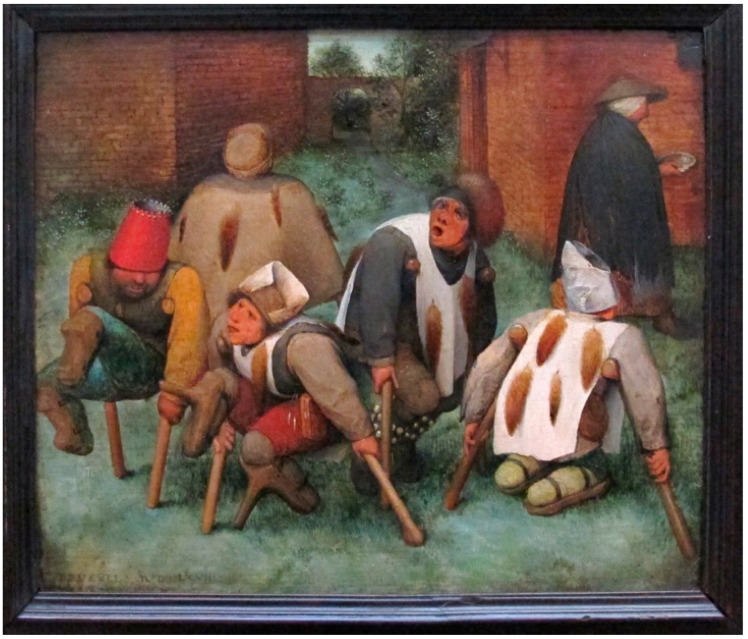
Bruegel’s *The Beggars* [79]. Note the fox tails indicating leprosy.

Although it is difficult to know with any certainty how disgust reactions have changed over recorded history, it is clear that disgust elicitors such as feces, urine, vomit, pus, and sick and dead people or animals were more frequently encountered in the past. Hunting was a widespread ancestral practice. It involved killing, skinning, dismembering, and removing the viscera from animals—as well as consuming many species including small rodents [74]. Prior to the privy, outside defecation and urination meant a high likelihood of encountering other people’s waste, or other people in the process of toileting—something that is still common in the developing world [75]. Exposure to dead and injured people or animals would also have been more frequent prior to segregated medical care, and disfiguring diseases like leprosy [76] (see Figure 2, which depicts a 16th-century characterization of the plight of those suffering from disease and infirmity). As exposure reduces disgust [76,77,78], our ancestors would likely have needed more potent disgust elicitors than we do.

In recorded history, there is evidence that death, disease, bodily products, and especially feces, generated disgust, and that this is so across cultures [76]. In Europe, and since the Middle Ages, three major changes appear to have occurred in relation to disgust. The first concerns the public expression of disgust, which has become gradually more inhibited. In the 16th century, educated people were advised that if they encountered something foul, they should not “… hold out the stinking thing for the other to smell… and say ‘I should like to know how much this stinks!’”. Instead, they were advised to ignore it, an approach that Miller [76] claims is now widely adopted in Western culture. The second change is that, with the advent of sewage systems, hospitals, and modern medicine, death, disease, feces, and other bodily products were encountered far less frequently than in the past. This absence of exposure may then have sensitized (relative to our ancestors) our contemporary disgust reactions to these elicitors. The third change is a notable broadening in the range of entities that can elicit disgust. While, in the Middle Ages, body odor was an inevitable consequence of not washing clothes and bodies, the advent of soap, readily available water for washing, and warm water made cleanliness an achievable goal for all. This change was largely driven by the manufacturers of soap and personal hygiene products via advertising, and they co-opted disgust towards what was previously the normal smell of unwashed humanity [80]. The consequence of all these changes is that, today, a person living in Europe will have little exposure to the type of disgust elicitors frequently encountered in the past. Moreover, they will have been trained since childhood to restrain their expression of disgust, and they will feel disgust towards things that would not have aroused this emotion in our ancestors (e.g., body odor, dirty fingernails, bad breath, etc.).

## 6. Conclusions

In conclusion, we have argued that the process model of disgust [9] provides the clearest framework for understanding the continuity of disgust in animals. Used together with the proposed disgust decision-tree criteria, which focus on ruling out alternate causes of avoidance such as fear or indifference, there is scope to establish the presence of pure, somatosensory, and anticipatory disgust in animals that have the capacity to experience nausea-associated gastrointestinal illness. Likewise, there is evidence that these forms of disgust are interlinked with the processes of conditioned taste aversions, and physical contamination. However, we expect that simulated disgust and the process of imagined contamination are likely exclusive to humans. Furthermore, we suggest that it is the capacity to experience simulated disgust and imagine contamination that has interacted with shifts in cultural attitudes towards cleanliness, culminating in the exaggerated levels of disgust evident in humans over the past centuries.

## Data Availability

Data sharing is not applicable as no new data were created or analyzed in this review.
